# Non-invasive preimplantation genetic testing for conventional IVF blastocysts

**DOI:** 10.1186/s12967-022-03596-0

**Published:** 2022-09-04

**Authors:** Pingyuan Xie, Shuoping Zhang, Yifang Gu, Bo Jiang, Liang Hu, Yue-qiu Tan, Yaxin Yao, Yi Tang, Anqi Wan, Sufen Cai, Yangyun Zou, Guangxiu Lu, Cheng Wan, Fei Gong, Sijia Lu, Ge Lin

**Affiliations:** 1 Hunan Normal University School of Medicine, Changsha, 410013 Hunan China; 2 NHC Key Laboratory of Human Stem Cell and Reproductive Engineering, School of Basic Medical Science, Institute of Reproductive and Stem Cell Engineering, Central South University, Changsha, 410008 Hunan China; 3 Hunan International Scientific and Technological Cooperation Base of Development and Carcinogenesis, Changsha, 410013 Hunan China; 4 Clinical Research Center for Reproduction and Genetics in Hunan Province, Reproductive and Genetic Hospital of CITIC-XIANGYA, Changsha, 410013 Hunan China; 5 National Engineering and Research Center of Human Stem Cells, Changsha, 410013 China; 6 Department of Clinical Research, Yikon Genomics Company, Ltd, Suzhou, 215000 China

**Keywords:** Spent culture medium, Non-invasive preimplantation genetic testing, Conventional IVF, Next generation sequencing

## Abstract

**Background:**

Previous studies suggested that non-invasive preimplantation genetic testing (niPGT) for intracytoplasmic sperm injection (ICSI) blastocysts can be used to identify chromosomal ploidy and chromosomal abnormalities. Here, we report the feasibility and performance of niPGT for conventional in vitro fertilization (IVF) blastocysts.

**Methods:**

This was a prospective observational study. In the preclinical stage, whole genome amplification and NGS were performed using the sperm spent culture medium (SCM). Then, trophectoderm (TE) biopsies and corresponding SCM derived from 27 conventional IVF monopronuclear embryos were collected. In the clinical stage, samples from 25 conventional IVF cycles and 37 ICSI cycles from April 2020–August 2021 were collected for performance evaluation.

**Results:**

Preclinically, we confirmed failed sperm DNA amplification under the current amplification system. Subsequent niPGT from the 27 monopronuclear blastocysts showed 69.2% concordance with PGT results of corresponding TE biopsies. In the clinical stage, no paternal contamination was observed in any of the 161 SCM samples from conventional IVF. While maternal contamination was observed in 29.8% (48/161) SCM samples, only 2.5% (4/161) samples had a contamination ratio ≥ 50%. Compared with that of TE biopsy, the performances of NiPGT from 161 conventional IVF embryos and 122 ICSI embryos were not significantly different (P > 0.05), with ploidy concordance rates of 75% and 74.6% for IVF and ICSI methods, respectively. Finally, evaluation of the euploid probability of embryos with different types of niPGT results showed prediction probabilities of 82.8%, 77.8%, 62.5%, 50.0%, 40.9% and 18.4% for euploidy, sex-chromosome mosaics only, low-level mosaics, multiple abnormal chromosomes, high-level mosaics and aneuploidy, respectively.

**Conclusions:**

Our research results preliminarily confirm that the niPGT approach using SCM from conventional IVF has comparable performance with ICSI and might broadening the application scope of niPGT.

**Supplementary Information:**

The online version contains supplementary material available at 10.1186/s12967-022-03596-0.

## Background

Preimplantation genetic testing for aneuploidy (PGT-A) using trophectoderm (TE) biopsy is currently widely used to identify euploid embryos. PGT-A has the clinical benefits of increasing the pregnancy rate, reducing the miscarriage rate and shortening the time required for pregnancy [1, 2]. However, this technique involves some invasive procedures, such as intracytoplasmic sperm injection (ICSI) and TE biopsy, which may adversely affect embryo development [3–5].

The discovery of DNA in blastocoele fluid (BF) and spent culture medium (SCM) from the embryo has led to the development of non-invasive PGT (niPGT). Numerous studies have demonstrated that niPGT is a potential means for embryo prioritization, although further investigations including genetic contamination, the optimization and standardization of culture conditions and medium retrieval protocols are required [6–10]. Compared to traditional methods, niPGT can avoid embryo biopsy damage, decrease the cost and require less micromanipulation.

In contrast to conventional in vitro fertilization (IVF), ICSI was originally used to treat couples with severe male factor infertility and was a “non-natural” process. Existing evidence indicates that children conceived through ICSI have an increased risk of chromosomal abnormalities, imprinting syndromes, autism, mental retardation, cancer and birth defects compared with naturally conceived children [11–14].

At present, although conventional IVF seems to be the most appropriate insemination method in non-male factor infertility, ICSI has been recommended for cases requiring PGT of embryos according to the American Society for Reproductive Medicine (ASRM) and European Society for Human Reproductive Embryology/Preimplantation Genetics Diagnosis International Society (ESHRE/PGDIS) [15, 16]. The rationale for ICSI use is to ensure monospermic fertilization and eliminate the possibility of contamination from sperm [17, 18]. For the same reason, niPGT is confined mainly to embryos derived from ICSI fertilization, which decreases accessibility for a wider patient population.

Recently, some studies found that sperm DNA fails to be amplified from TE samples under whole-genome amplification (WGA) conditions, bringing hope regarding the use of conventional IVF in PGT and niPGT in non-male factor infertility [19]. The ASRM in 2020 suggested that in PGT cycles without male factor infertility, ICSI should be limited to cases where sperm contamination could affect the accuracy of test results. More in-depth analysis should be performed to confirm the feasibility of using conventional IVF in niPGT cycles [15]. Therefore, in the present study, first, WGA and NGS were performed using sperm samples and SCM collected from the zona pellucida with sperm. Then, parental contamination was evaluated in SCM from conventional IVF injection. Furthermore, the performance of niPGT using SCM from IVF and ICSI blastocysts was compared to investigate the feasibility of niPGT for prioritizing embryos from conventional IVF.

## Methods

### Ethics statement

This study was approved by the ethical committee of the CITIC-Xiangya Reproductive & Genetic Hospital (LL-SC-2020–006). Written informed consent was obtained from all study participants.

### Sample preparation

All samples were provided by the Reproductive & Genetic Hospital of CITIC-Xiangya from April 2020 to August 2021. To detect sperm-derived DNA in culture medium, different numbers (n = 3, 10, 50, 100) of donor sperm were placed in 25 μl blastocyst culture medium (Cook Medical, Brisbane, Australia) and cultured for different lengths of time (4–8 h, 1 day, 3 days and 5 days) at 37 °C under 6% CO_2_, 5% O_2_ and 89% N_2_. Each group was replicated four times. In addition, considering the structural change to sperm after the acrosome reaction, seven zona pellucidas (ZPs) were collected by completely removing the blastomeres and fragmentations in IVF-inseminated arrested embryos and cultured in 25 μl medium for 5 days. Then, the ZPs were removed, and the SCM were collected.

In preclinical testing, to evaluate the effect of conventional insemination on the results of niPGT, we included 27 blastocysts derived from 74 monopronuclear (1PN) zygotes in conventional IVF cycles. On Day 3, the 1PN embryos were transferred to blastocyst medium, and a change in fresh medium (each embryo per 25 μl droplet) combined with complete cumulus cell removal was performed on Day 4. Blastocyst morphology was assessed on Day 5 according to the Gardner grading criteria and again on Day 6 if they failed to meet the biopsy criteria on Day 5. TE biopsy was performed if the embryo reached a morphologic grade of at least 4BC, and the corresponding SCM were collected simultaneously.

To determine the effect of parental contamination ratio on NICS CNV calling, We collected fetal genomic DNA extracted from tissue of spontaneous abortion (46, XN, -8p (× 1), + 8q (× 3, mos, ~ 60%)) and parents genomic DNA extracted from peripheral blood.

**Fig. 1:**
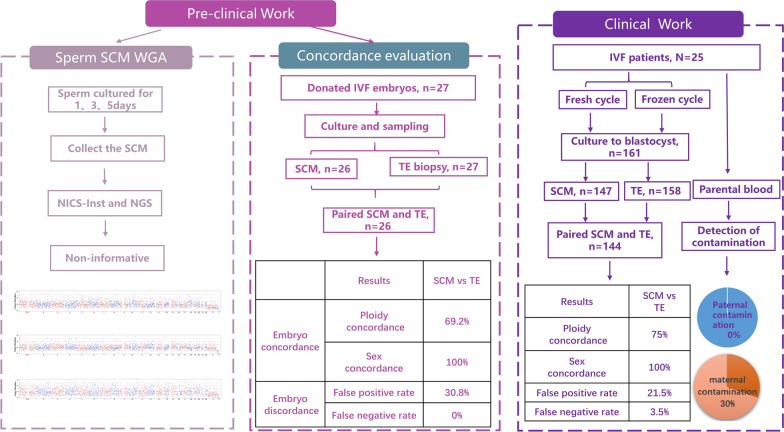
Illustration of the systematic validation of the IVF–NICS assay. Two parts were included. The left panel shows the clinical work, which includes the sperm culture medium WGA and NICS detection and concordance evaluation. The right panel shows clinical work, and a total of 161 embryos were assessed using both the NICS and TE biopsy samples from 25 IVF patients

During clinical testing, 15 fresh cycles and 10 frozen cycles using conventional IVF were included, and parental blood samples were collected to identify parental contamination in SCM. All fresh or thawed Day (D)3 embryos were cultured to blastocysts for PGT-A and niPGT, and the procedures of embryo culture, TE biopsy and SCM collection were the same as those described for preclinical testing. To assess the validity of niPGT for IVF versus ICSI insemination cycles, the data from PGT-A combined with niPGT cycles inseminated by ICSI at the same time period in our centre were collected (Fig. 1).

### Whole-genome amplification (WGA) and sequencing

WGA was performed using SCM from sperm and blastocysts, or TE biopsy, followed by library preparation using NICSInst (Yikon Genomics; EK100100724 NICS-Inst Library Preparation Kit) [20, 21]. To analyse the ploidy status of blastocysts, the library of SCM or TE samples was sequenced using a NextSeq 550 sequencer (Cat No. SY-415–1002, Illumina, Inc., USA) with a single-ended read length of 55 bp. Approximately 2 million raw reads were generated for each sample. CNVs for all chromosomes were analysed to determine the euploidy or aneuploidy status of each embryo [20, 21]. Aneuploid embryos were diagnosed when the extent of mosaicism was above 30% and the segmental aneuploidy was greater than 4 Mb.

For biopsies, whole-chromosome aneuploidies, mosaicism (between 30%–70%), and segmental aneuploidies (deletion or duplication > 4 Mb) were identified.

### Genotyping assay of SCM and parental genomes

The Infinium Asian Screening Array (ASA) bead chip (Cat No. 20016317, Illumina, Inc., USA) and the iScan system (Cat No. SY-101–1001, Illumina, Inc., USA) were used to determine the genotype of each SCM and parental genome [22]. Parental genomic DNA was extracted from the parental peripheral blood. Genomic DNA, along with the amplified DNA of SCM samples, was linearly amplified, fragmented, precipitated and hybridized according to the manufacturer’s instructions. Signal scanning was performed with the iScan system. The genotype of each sample was analysed with GenomeStudio software (version 2.0, Illumina, Inc., USA), and the B allele frequency and log R ratio (LRR) values of all single nucleotide polymorphism (SNPs) were generated simultaneously. The quantification parental contamination testing (qPCT) model can effectively detect the risk of parent DNA contamination in samples based on allelic ratio analysis by using sequencing results of whole-genome amplification products. The detailed method for qPCT model has been described in the preprint article [22].

### Statistical analysis

Data analyses were conducted using SPSS version 25 (IBM, Armonk, NY, USA). Categorical data are expressed as counts and percentages and were determined to be statistically significant using the chi-square test or Fisher’s exact test. A P-value (two-sided) equal to or less than 0.05 was considered statistically significant.

## Results

As shown in Fig. 1, two parts were included. The first part is the preclinical work. The first stage is the sperm SCM WGA and NGS and then the concordance evaluation between the TE and SCM of IVF monopronuclear embryos. The second part is the clinical work, including the detection of parental contamination of SCM and the performance of NiPGT using SCM from IVF and ICSI blastocysts.

### Preclinical work

First, to determine whether the sperm would bring paternal contamination, WGA and NGS were performed using the SCM of sperm and ZPs. Following NGS, all the samples generated an amplification-failure (AF) pattern, similar to the blank media samples. This result suggested that sperm DNA fails to amplify under the current amplification system, which provides the possibility for the application of NiPGT using SCM from conventional IVF (Additional file 2: Tables S1, S2).

In the second stage, a total of 27 donated monopronuclear embryos from conventional IVF were used to evaluate the performance of NiPGT. Two different samples from the same embryo, including the TE and SCM, were collected separately and used for subsequent sequencing. One NiPGT sample was an AF, so the CNV results from 26 TE and NiPGT were graphed and compared. As shown in Table 1, Additional file 2: Table S3, Table S4, using the TE as the gold standard, the NiPGT showed a 69.2% concordance rate, similar to the reference results using SCM from ICSI [23, 24]. This result indicated that the SCM from conventional IVF may also be used for niPGT.

**Table 1:** The performance of NiPGT in 27 donated embryos using TE as the gold standard

Performance of SCM	Results	95% CI
WGA success rate (SCM)	96.3% (26/27)	81.7–99.8
WGA success rate (TE)	100.0% (27/27)	87.5–100
Ploidy concordance	69.2% (18/26)	50.0–83.5
Sex concordance	100.0% (26/26)	87.1–100
Sensitivity	100.0% (7/7)	64.6–100
Specificity	57.9% (11/19)	36.3–76.9
Positive predictive value (PPV)	46.7% (7/15)	24.8–69.9
Negative predictive value (NPV)	100% (11/11)	74.1–100
False positive rate	30.8%(8/26)	16.5–50.0
False negative rate	0%(0/26)	0–12.9

### Parental contamination analysis of SCM

**Fig. 2:**
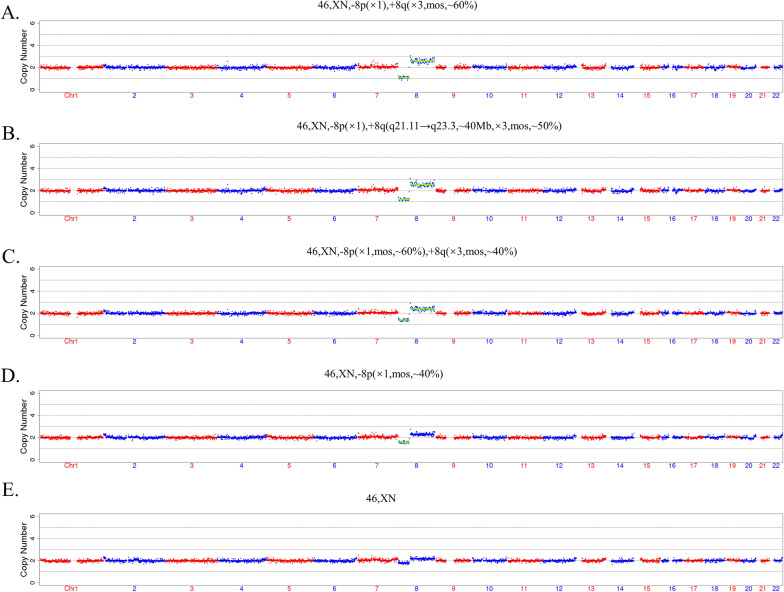
CNV results of spontaneous abortions mixed with blood samples from parents in specific proportions. (a) The CNV result is 46,XN,-8p(× 1), + 8q(× 3,mos, ~ 60%) of the spontaneous abortions. (b–e) Spontaneous abortions mixed with different proportions (10%, 30%, 50%, 70%) of parental blood samples. The CNV results are 46,XN, -8p(× 1), + 8q(q21.11 → q23.3, ~ 40 Mb, × 3,mos, ~ 50%); 46,XN, -8p(× 1,mos, ~ 60%), + 8q(× 3,mos, ~ 40%); 46,XN, -8p(× 1,mos, ~ 40%); 46,XN, respectively

To further address the issue of the quantification of parental contamination and confirming the effect of parental contamination at different level on CNV interpretation, genome DNA (gDNA) from a spontaneous abortion sample and gDNA from parents blood samples were mixed in specific proportions (10%, 30%, 50%, 70%). The CNV-seq results of the spontaneous abortion sample was 46,XN,-8p(× 1), + 8q(× 3,mos, ~ 60%). The mixed gDNA was diluted to an approximate concentration of 50 pg/μL and then amplified, sequenced and analyzed in accordance with the PGT-A procedure. The qPCT model was applied to analysis parental contamination of SCM [22]. As a result, the CNV results of abortion gDNA showed a positive correlation with the proportion of mixed parental gDNA (Fig. 2b–e). The relative quantification of parental contamination could be performed by using the standard curve. While the proportion of parental contamination was 30%, the 8q abnormality was detected, and the CNV result was 46, XN, 8p(× 1,mos, ~ 60%), + 8q(× 3,mos, ~ 40%) (Fig. 2c). However, we found the CNV abnormality calling was affected when the proportion of parental contamination reached more than 50%, the CNV result was 46,XN, -8p(× 1,mos, ~ 40%) and 46, XN, respectively (Fig. 2d, e).

Based on the qPCT model established in our preprint article [22], in the clinical application stage, first, parental contamination was analysed for the SCM of 161 blastocysts from 25 conventional IVF cycles. Among the 161 SCM samples, no paternal contamination was observed in any of the SCM samples (Table 2 and Additional file 1: Figure S1). These results further confirmed that sperm would not affect the NiPGT results (Additional file 3: Table S5).

**Table 2:** Analysis of parental contamination in SCM in IVF cycles

Result	SCM
Detection success rate	98.1% (158/161)
No maternal contamination	69.6% (110/158)
Maternal contamination (ratio < 30%)	16.5% (26/158)
Maternal contamination (30% ≤ ratio < 50%)	11.4% (18/158)
Maternal contamination (50% ≤ ratio < 70%)	1.9% (3/158)
Maternal contamination (ratio ≥ 70%)	0.6% (1/158)
No paternal contamination	100% (158/158)
Paternal contamination	0% (0/158)

Maternal contamination was observed in 48 SCM samples. Among the 48 SCM samples, the contamination ratio was < 30% in 26 samples, between 30–50% in 18 samples and ≥ 50% in 4 samples (Table 2). Furthermore, compared to that of fresh blastocysts, the maternal contamination ratio was lower in samples from cryopreserved blastocysts (19.2% vs. 35.8%, P = 0.033). The results are shown in Additional file 2: Table S6.

### The performance of NiPGT using SCM from IVF and ICSI blastocysts

To evaluate whether the insemination method impacts the NiPGT results, we compared the performance of NiPGT from IVF and ICSI blastocysts. A total of 161 samples from 25 conventional IVF cycles and 122 samples from 37 ICSI cycles were used to investigate the performance between trophectoderm biopsy DNA and SCM. The details of the clinical baseline characteristics are presented in Additional file 2: Table S7.

First, the amplification failure rate of was observed in 8.7% (14/161) and 3.3% (4/122) of embryonic cfDNA samples from conventional IVF and ICSI, respectively (Table 3; Additional file 3: Tables S5 and Additional file 4: S8). Comparison of the results between groups according to embryo quality and exposure time revealed that the amplification success rates of the good, fair and poor embryo groups were 82.4%, 95.2% and 95.8%, respectively, and that the amplification success rates of the D5, D6 and D7 embryo groups were 81.0%, 92.0% and 100.0%, respectively (Additional file 2: Tables S9, S10).

**Table 3:** The NICS performance of IVF versus ICSI in detecting chromosomal abnormalities

Assay (n)	NICS amplification success rate, % (95% CI)	TE amplification success rate, % (95% CI)	Embryo	Assay (n)	Concordance, % (95% CI)	Sensitivity, % (95% CI)	Specificity, % (95% CI)	PPV, % (95% CI)	NPV, % (95% CI)
IVF (161)	91.3 (85.9–94.7)	98.1 (94.7–99.5)	Original	IVF (144)	62.5 (54.4–70.0)	93.0 (84.6–97.0)	32.9 (23.2–44.3)	57.4 (48.3–66.1)	82.8 (65.5–92.4)
ICSI (122)	96.7 (91.9–98.7)	100 (96.9–100)		ICSI (118)	67.0 (58.0–74.8)	86.2 (75.7–92.5)	43.4 (31.0–56.7)	65.1 (54.6–74.4)	71.9 (54.6–84.4)
p value	0.064	0.352		p value	0.45	0.19	0.23	0.27	0.31
			Adjusted	IVF-Adjusted (144)	75.0 (67.3–81.4)	91.4 (81.4–96.3)	64.0 (53.4–73.3)	63.1 (52.4–72.6)	91.7 (81.9–96.4)
				ICSI-Adjusted (118)	74.6 (66.0–81.6)	84.2 (72.6–91.5)	65.6 (53.1–76.3)	69.6 (57.9–79.2)	81.6 (68.6–90.0)
				p value	0.94	0.24	0.84	0.40	0.12

Original: TE size ≥ 4 Mb, 30% mosaic cut-off value; NICS whole chromosome, 30% mosaic cut-off value. Adjusted: TE size ≥ 4 Mb, 50% mosaic cut-off value; NICS whole chromosome, 50% mosaic cut-off value

Then, we treated the results from TE biopsy as the gold standard to evaluate the performances of NiPGT from IVF and ICSI blastocysts. We considered the results concordant if the two assays both generated chromosomal normal or chromosomal abnormal results. As shown in Table 3, compared with the TE biopsy,

IVF–NiPGT vs. ICSI–NiPGT yielded a performance of 93.0% (84.6–97.0) vs. 86.2% (75.7–92.5), 32.9% (23.2–44.3) vs. 43.4% (31.0–56.7), 57.4% (48.3–66.1) vs. 65.1% (54.6–74.4), and 82.8% (65.5–92.4) vs. 71.9% (54.6–84.4) for sensitivity, specificity, PPV, and NPV, respectively. The IVF and ICSI samples were 62.5% (54.4–70.0) and 67.0% (58.0–74.8) concordant with the corresponding TE biopsy samples. Comparatively, the performances of NiPGT from conventional IVF and ICSI were not statistically significant (Table 3, P > 0.05). Furthermore, we compared the results under a 50% mosaic cut-off value. Under the 50% mosaic cut-off value, the concordance, sensitivity, specificity, PPV and NPV of the two insemination methods were not different (P = 0.94, P = 0.24, P = 0.84, P = 0.40, P = 0.12, respectively) (Table 3). The concordance rate increased to 75% and 74.6% for the IVF and ICSI methods, respectively. In conclusion, no significant differences were observed between the two groups (Table 3, P > 0.05).

### Categorizing and prioritizing embryos according to NiPGT and TE–PGT results

**Fig. 3:**
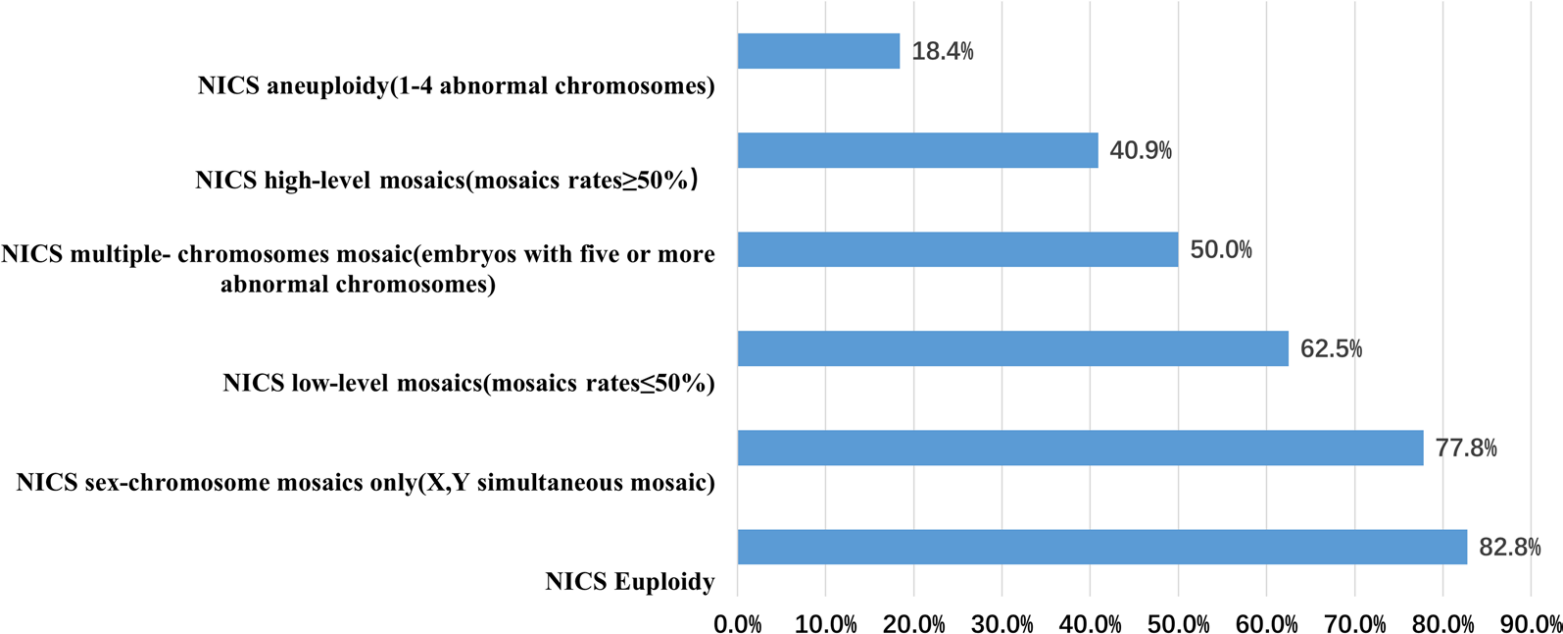
NICS results of different groups predicting a different ploidy normal probability in the TE biopsies

We further evaluated the euploid probability of embryos with different NiPGT aneuploidy results. The CNV results from SCM can be classified into five different categories: (1) euploidy, (2) sex-chromosome mosaics only, (3) low-level mosaics (mosaic rates ≤ 50%), multiple abnormal chromosomes (embryos with five or more abnormal chromosomes), high-level mosaics (mosaic rates ≥ 50%), and aneuploidy (1–4 abnormal chromosomes). Similarly, the results from TE can be classified into three different categories: euploidy, mosaic and aneuploidy (Table 4). We noted that based on the NiPGT results of the five groups, each predicted a different ploidy normal probability in the TE biopsies. The predicted euploid probabilities were 82.8%, 77.8%, 62.5%, 50.0%, 40.9% and 18.4%, as shown in Fig. 3.

**Table 4:** Different NICS results corresponding to TE results

	TE		
	Euploidy	Mosaics only	Aneuploidy
NICS			
Euploidy	82.8%(24/29)	13.8% (4/29)	3.5% (1/29)
Sex-chromosome mosaics only (X,Y simultaneous mosaic)	77.8% (7/9)	22.2% (2/9)	0% (0/9)
Low-level mosaics (mosaics rates ≤ 50%)	62.5% (15/24)	20.8% (5/24)	16.7% (4/24)
Multiple abnormal chromosomes (embryos with five or more abnormal chromosomes)	50.0% (11/22)	9.1% (2/22)	40.9% (9/22)
High-level mosaics (mosaics rates ≥ 50%)	40.9% (9/22)	18.2% (4/22)	40.9% (9/22)
Aneuploidy (1–4 abnormal chromosomes)	18.4% (7/38)	7.9% (3/38)	71.1% (28/38)

## Discussion

Our study found that NiPGT from conventional IVF blastocysts exhibited high concordance rates (75%) between embryonic cfDNA and corresponding TE biopsies, which was similar to recent publications indicating concordance rates from ICSI blastocysts [23–25]. These results bring hope for the application of NiPGT for prioritizing embryos using SCM from conventional IVF. Moreover, the parental contamination analysis of the SCM indicated that sperm would not affect the results under the amplification used in the current study.

In the preclinical work stage, we confirmed that no sperm DNA amplification product was observed under the NICSInst method, even if 100 sperm cells or ZPs with sperm were cultured for 5 days in embryo culture medium. The reason may be due to the need to collect 10 µl SCM for amplification, but the volume of lysate was 5 µl. The culture medium diluted the lysate, resulting in the originally mild lysis conditions of NICSInst amplification conditions not being sufficient to amplify sperm DNA. It has recently been shown that sperm DNA fails to amplify under the PicoPLEX technology used for PGT-A on TE samples [19]. In fact, sperm DNA amplification requires strong lysis conditions to achieve amplification, and the WGA lysis conditions are milder, which can effectively amplify biopsy cells (polar bodies, blastomeres, or trophoblast cells) without amplifying sperm DNA. Paternal cell contamination resulting from IVF may not interfere with PGT, a concern that was reported previously [15, 19]. Therefore, the WGA technology used in this study can prevent sperm contamination caused by IVF fertilization from causing substantial interference with PGT detection. The ASRM consensus also proposes that once the technology is improved to the extent that the interference of paternal cells is indeed negligible, IVF fertilization may be more useful in PGT patients with normal sperm count, motility, and morphology [15]. The results of this study reassure that conventional IVF can be safely applied in niPGT and PGT for couples with non-male factor infertility.

Data on the performances of NiPGT between conventional IVF and ICSI showed no significant difference (concordance: 75.0% versus 74.6%; sensitivity: 91.4% versus 84.2%; specificity: 64.0% versus 65.6%; Table 3). This is similar to the results of a recent prospective study, which suggested that embryonic cell-free DNA from IVF and ICSI techniques had similar sensitivity (87.9% vs. 80.9%) and specificity (69.9% vs. 78.6%) [24]. The reason may be that the ICSI and IVF techniques have no effect on embryo development and the probability of euploidy. In previous studies, De Munck et al. [26] compared the developmental ability and euploidy of patients with non-male infertility after conventional IVF or ICSI sister oocyte fertilization. There was no significant difference between IVF and ICSI in terms of the fertilization rate, embryo development or the number of euploid embryos. A retrospective study by Deng et al. [27] found that the numbers of IVF–PGT-A and ICSI–PGT-A had similar embryo aneuploidy and mosaic ratios. Conventional IVF fertilization is unlikely to cause serious contamination during PGT-A. Routine IVF fertilization is recommended as the preferred method of insemination in the PGT-A cycle, but ICSI is more applicable to cases of male infertility. Our data also showed that there was no difference between IVF and ICSI in terms of the proportion of euploid embryos (59.5% vs. 55.9%, P = 0.553, Additional file 2: Table S11).

Maternal contamination is an important technical challenge for the clinical application of NICS, which affected the accuracy of NICS results. Usually, cumulus cells are removed by enzymatic methods combined with mechanical methods before ICSI, but they are impossible to remove completely. One study suggested that approximately one-third of the samples from ICSI had a ratio greater than 60% [28]. In artificially contaminated experiments, our results suggested the higher the parental contamination ratio, the greater the impact, especially when the contamination ratio exceeded 50%, false negative results may be obtained.Our results from IVF indicated that only 2.5% (4/158) of samples had a ratio greater than 50%. This may be due to the sampling method used for the embryos. We removed the embryos again on D3 and transferred the embryo into culture medium after noon on D4 and then cultured it until D6, as described in a previous article [29]. Refer to avoid and correct maternal contamination, First, developing optimized sample collection method to reduce maternal contamination such as change the embryo medium at day 3 and day 4, simultaneously, careful denudation of surrounding cumulus cells before medium change. Second, based on the difference between embryonic and maternal DNA, we hope to develop corresponding detection platform such as RNA-Seq, methylation-Seq and bioinformatics algorithms to distinguish the maternal DNA and embryonic DNA is also encouraged.

Our study indicated that the discordant results were caused mainly by false positives in the NiPGT. “False positive” samples should reflect mainly CNV mosaicism in SCM cfDNA. This may be related to the self-correction of embryonic development. A study in mice and rhesus monkeys suggested that aneuploid cells in embryos are eliminated by apoptosis, cellular fragmentation and blastomere exclusion. Based on this, increasing the mosaicism threshold may improve the concordance rate by minimizing the risk of false positives. In previous research, different studies have different reporting standards for the mosaic ratio, ranging from 30 to 60%. In this study, the raw concordance rate between SCM and TE biopsies was 62.5% in terms of overall ploidy when we set the mosaicism identification threshold to 30%; however, the concordance rate was increased to 75%. This is due mainly to the decrease in the false positive rate.

DNA AF is a common concern in NiPGT. In this study, the detection failure (AF and inconclusive result) rate was 8.7% with 25-μl media droplets. Rubio et al. reported successful DNA amplification in 97.4% (1,267/1,301) of samples with 10-μl media droplets [24]. Additionally, Kuznyetsov et al. [30] reported a 100% DNA amplification success rate from all 47 samples using a combination of blastocyst culture media and blastocoel fluid. Furthermore, we may improve the amplification success rate by decreasing the media droplet volume and releasing blastocoel fluid.

The amplification success rate of the good embryo groups was 82.4%, which was lower than the 95.2% and 95.8% success rates of the fair and poor embryo groups, respectively. Moreover, the D5 (81.0%) embryo group had the lowest amplification success rate compared with the D6 (92.0%) and D7 (100.0%) embryo groups (Additional file 2: Tables S9–S11). These results suggested that the amplification success rate was decreased in good-quality embryos and D5 blastocysts. In addition, the amplification success rate may be negatively correlated with euploidy and clinical outcomes. In 2019, Magli et al. [31] showed that compared with that of aneuploid blastocysts (n = 150, 81%), the amplification success rate of blastocoel fluid from euploid blastocysts was significantly reduced (n = 32, 45%). The clinical pregnancy rate of the BF amplification failure group was 77%, but that of the BF amplification success group was 37%. This is also consistent with our results that embryo morphology is better, blastocyst formation occurs earlier, and the amplification success rate is lower. In the future, the concentration of original cfDNA may be used as one of the indicators for predicting clinical outcome.

We also evaluated the euploid probability of embryos with different NiPGT aneuploidy results, so we propose to transfer embryos following the order from high euploid rate to low euploid rate (Table 4 and Fig. 3), as follows: euploid > sex-chromosome mosaics > low-level mosaics > multiple abnormal chromosomes > high-level mosaics > aneuploidy (euploid rate: 82.8%, 77.8%, 62.5%, 50.0%, 40.9%, 18.4%, respectively). The result of NiPGT enables one to judge the potential of embryo implantation and may be used as a reference factor for the order of embryo implantation, to avoid wasting embryos due to false positivity. In addition, this result is similar to the embryo selection strategy mentioned in a previous article [32]. In this article, Chen et al. established an embryo selection strategy by evaluating the probability of euploidy using culture medium to avoid wasting embryos.

In conclusion, our research results preliminarily confirmed that the NiPGT approach using SCM from conventional IVF as well as ICSI can be used to prioritize embryos, which broadens the application scope of NiPGT. Compared to the invasive method, the NiPGT approach has important advantages, such as avoiding biopsy and decreasing cost. Especially for patients who are undergoing PGT-A for non-male factors, non-invasive strategies of combined IVF and NiPGT can be considered the preferred option.

## Supplementary Information

**Additional file 1: Figure S1.** Proportion of parental contamination in SCM in IVF cycles. A: The proportion of maternal contamination. B: The proportion of paternal contamination.

**Additional file 2: Table S1.** Sperm CNV results of nonlysis group. **Table S2.** Sperm CNV results of lysis group. **Table S3.** CNV results of SCM and TE of samples with abnormal fertilization **Table S4.** The overview of NGS results from SCM and TE in 27 donated 1PN embryos. **Table S6.** Comparison of maternal contamination between fresh embryos and frozen embryos. **Table S7.** Clinical baseline characteristics of patients. **Table S9.** Comparison of the diagnostic parameters of SCM from different blastocyst quality and different sampling times in 161 clinical IVF samples. **Table S10.** The TE results of 9 ‘Good’ samples. **Table S11.** The proportion of euploid embryos between the IVF and ICSI groups.

**Additional file 3: Table S5.** The CNV results, parental contamination and other information forms of 161 IVF samples.

**Additional file 4: Table S8.** The CNV results and other information forms of 122 ICSI samples.

## Data Availability

The datasets used and/or analysed during the current study are available from the corresponding author on reasonable request.

## Abbreviations

niPGT: Non-invasive preimplantation genetic testing
SCM: Spent culture media
TE: Trophectoderm
ICSI: Intracytoplasmic sperm injection
WGA: Whole-genome amplification
ZPs: Zona pellucidas
1PN: Monopronuclear
NICS: Noninvasive chromosomal screening
cfDNA: Cell free DNA
BF: Blastocoele fluid
AF: Amplification failure
qPCT: Quantification parental contamination testing
