# Supplementary figures and images for: Non-invasive preimplantation genetic testing for conventional IVF blastocysts
Source: J Transl Med. 2022 Sep 4;20:396. doi: 10.1186/s12967-022-03596-0 (PMC9441092; doi:10.1186/s12967-022-03596-0)

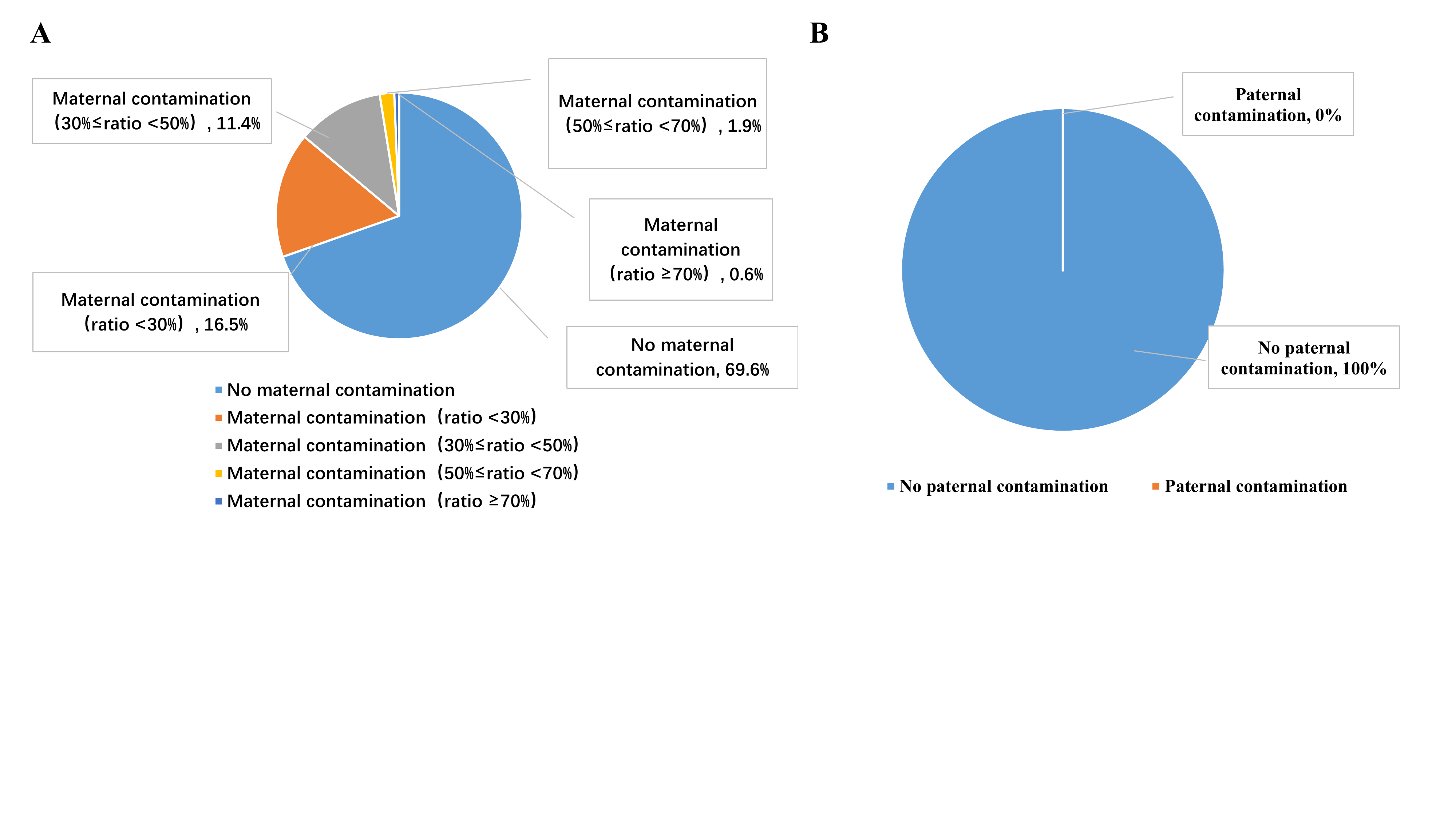

Supplement: Supplementary file 1 — Additional file 1: Figure S1. Proportion of parental contamination in SCM in IVF cycles. A: The proportion of maternal contamination. B: The proportion of paternal contamination. [file 12967_2022_3596_MOESM1_ESM.tif]
