# Supplementary material for: Non-invasive preimplantation genetic testing for conventional IVF blastocysts
Source: J Transl Med. 2022 Sep 4;20:396. doi: 10.1186/s12967-022-03596-0 (PMC9441092; doi:10.1186/s12967-022-03596-0)
Supplement: Supplementary file 2 — Additional file 2: Table S1. Sperm CNV results of nonlysis group. Table S2. Sperm CNV results of lysis group. Table S3. CNV results of SCM and TE of samples with abnormal fertilization Table S4. The overview of NGS results from SCM and TE in 27 donated 1PN embryos. Table S6. Comparison of maternal contamination between fresh embryos and frozen embryos. Table S7. Clinical baseline characteristics of patients. Table S9. Comparison of the diagnostic parameters of SCM from different blastocyst quality and different sampling times in 161 clinical IVF samples. Table S10. The TE results of 9 ‘Good’ samples. Table S11. The proportion of euploid embryos between the IVF and ICSI groups. [file 12967_2022_3596_MOESM2_ESM.docx]

**Supplementary materials**

**Supplementary Table 1.** Sperm CNV results of nonlysis group

| **Sample type** | **Sperm count (piece)** | **Repeat** | **Library concentration (ng/μl)** | **CNV_results** |
| --- | --- | --- | --- | --- |
| medium containing sperm (cultured 4–8 h) | 3 | 1 | 0.19 | N/A |
| -medium containing sperm (cultured 4–8 h) | 10 | 1 | 0.192 | N/A |
| medium containing sperm (cultured 4–8 h) | 50 | 1 | 0.178 | N/A |
| medium containing sperm (cultured 4–8 h) | 100 | 1 | 0.248 | N/A |
| medium containing sperm (cultured 4–8 h) | 3 | 2 | 0.182 | N/A |
| medium containing sperm (cultured 4–8 h) | 10 | 2 | 0.236 | N/A |
| medium containing sperm (cultured 4–8 h) | 50 | 2 | 0.222 | N/A |
| medium containing sperm (cultured 4–8 h) | 100 | 2 | 0.152 | N/A |
| medium containing sperm (cultured 4–8 h) | 3 | 3 | 0.294 | N/A |
| medium containing sperm (cultured 4–8 h) | 10 | 3 | 0.192 | N/A |
| medium containing sperm (cultured 4–8 h) | 50 | 3 | 0.2 | N/A |
| medium containing sperm (cultured 4–8 h) | 100 | 3 | 0.166 | N/A |
| medium containing sperm (cultured 4–8 h) | 3 | 4 | 0.294 | N/A |
| medium containing sperm (cultured 4–8 h) | 10 | 4 | 0.198 | N/A |
| medium containing sperm (cultured 4–8 h) | 50 | 4 | 0.246 | N/A |
| medium containing sperm (cultured 4–8 h) | 100 | 4 | 0.216 | N/A |
| medium containing sperm (cultured 1 d) | 3 | 1 | 0.25 | N/A |
| medium containing sperm (cultured 1 d) | 10 | 1 | 0.274 | N/A |
| medium containing sperm (cultured 1 d) | 50 | 1 | 0.198 | N/A |
| medium containing sperm (cultured 1 d) | 100 | 1 | 0.226 | N/A |
| medium containing sperm (cultured 1 d) | 3 | 2 | 0.222 | N/A |
| medium containing sperm (cultured 1 d) | 10 | 2 | 0.254 | N/A |
| medium containing sperm (cultured 1 d) | 50 | 2 | 0.23 | N/A |
| medium containing sperm (cultured 1 d) | 100 | 2 | 0.238 | N/A |
| medium containing sperm (cultured 1 d) | 3 | 3 | 0.322 | N/A |
| medium containing sperm (cultured 1 d) | 10 | 3 | 0.264 | N/A |
| medium containing sperm (cultured 1 d) | 50 | 3 | 0.252 | N/A |
| medium containing sperm (cultured 1 d) | 100 | 3 | 0.23 | N/A |
| medium containing sperm (cultured 1 d) | 3 | 4 | 0.252 | N/A |
| medium containing sperm (cultured 1 d) | 10 | 4 | 0.214 | N/A |
| medium containing sperm (cultured 1 d) | 50 | 4 | 0.266 | N/A |
| medium containing sperm (cultured 1 d) | 100 | 4 | 0.266 | N/A |
| medium containing sperm (cultured 3 d) | 3 | 1 | 0.226 | N/A |
| medium containing sperm (cultured 3 d) | 10 | 1 | 0.214 | N/A |
| medium containing sperm (cultured 3 d) | 50 | 1 | 0.258 | N/A |
| medium containing sperm (cultured 3 d) | 100 | 1 | 0.182 | N/A |
| medium containing sperm (cultured 3 d) | 3 | 2 | 0.24 | N/A |
| medium containing sperm (cultured 3 d) | 10 | 2 | 0.212 | N/A |
| medium containing sperm (cultured 3 d) | 50 | 2 | 0.218 | N/A |
| medium containing sperm (cultured 3 d) | 100 | 2 | 0.264 | N/A |
| medium containing sperm (cultured 3 d) | 3 | 3 | 0.22 | N/A |
| medium containing sperm (cultured 3 d) | 10 | 3 | 0.202 | N/A |
| medium containing sperm (cultured 3 d) | 50 | 3 | 0.356 | N/A |
| medium containing sperm (cultured 3 d) | 100 | 3 | 0.212 | N/A |
| medium containing sperm (cultured 3 d) | 3 | 4 | 0.256 | N/A |
| medium containing sperm (cultured 3 d) | 10 | 4 | 0.288 | N/A |
| medium containing sperm (cultured 3 d) | 50 | 4 | 0.244 | N/A |
| medium containing sperm (cultured 3 d) | 100 | 4 | 0.284 | N/A |
| medium containing sperm (cultured 5 d) | 3 | 1 | 0.282 | N/A |
| medium containing sperm (cultured 5 d) | 10 | 1 | 0.242 | N/A |
| medium containing sperm (cultured 5 d) | 50 | 1 | 0.192 | N/A |
| medium containing sperm (cultured 5 d) | 100 | 1 | 0.186 | N/A |
| medium containing sperm (cultured 5 d) | 3 | 2 | 0.31 | N/A |
| medium containing sperm (cultured 5 d) | 10 | 2 | 0.286 | N/A |
| medium containing sperm (cultured 5 d) | 50 | 2 | 0.188 | N/A |
| medium containing sperm (cultured 5 d) | 100 | 2 | 0.284 | N/A |
| medium containing sperm (cultured 5 d) | 3 | 3 | 0.312 | N/A |
| medium containing sperm (cultured 5 d) | 10 | 3 | 0.332 | N/A |
| medium containing sperm (cultured 5 d) | 50 | 3 | 0.24 | N/A |
| medium containing sperm (cultured 5 d) | 100 | 3 | 0.25 | N/A |
| medium containing sperm (cultured 5 d) | 3 | 4 | 0.254 | N/A |
| medium containing sperm (cultured 5 d) | 10 | 4 | 0.276 | N/A |
| medium containing sperm (cultured 5 d) | 50 | 4 | 0.274 | N/A |
| medium containing sperm (cultured 5 d) | 100 | 4 | 0.276 | N/A |
| sperm injection zona pellucida | / | 1 | 0.246 | N/A |
| sperm injection zona pellucida | / | 2 | 0.238 | N/A |
| sperm injection zona pellucida | / | 3 | 0.254 | N/A |
| sperm injection zona pellucida | / | 4 | 0.23 | N/A |
| sperm injection zona pellucida | / | 5 | 0.27 | N/A |
| In vitro fertilization zona pellucida | / | 1 | 0.3 | N/A |
| In vitro fertilization zona pellucida | / | 2 | 0.26 | N/A |
| In vitro fertilization zona pellucida | / | 3 | 0.228 | N/A |
| In vitro fertilization zona pellucida | / | 4 | 0.222 | N/A |
| In vitro fertilization zona pellucida | / | 5 | 0.2 | N/A |
| In vitro fertilization zona pellucida | / | 6 | 0.172 | N/A |
| In vitro fertilization zona pellucida | / | 7 | 0.174 | N/A |
| Blank drop | / | 1 | 0.274 | N/A |
| Blank drop | / | 2 | 0.244 | N/A |
| Blank drop | / | 3 | 0.198 | N/A |
| Blank drop | / | 4 | 0.26 | N/A |
| Positive control 10 pg GM12878 gDNA | / | 1 | 17.8 | 46,XN |
| Positive control 10 pg GM12878 gDNA | / | 2 | 14.2 | 46,XN |

**Supplementary Table 2.** Sperm CNV results of lysis group

| **Sample type** | **Sperm count** | **Repeat** | **Library concentration (ng/μl)** | **CNV_results** |
| --- | --- | --- | --- | --- |
| medium containing sperm (cultured 4–8 h) | 3 | 1 | 0.134 | N/A |
| medium containing sperm (cultured 4–8 h) | 10 | 1 | 0.136 | N/A |
| medium containing sperm (cultured 4–8 h) | 50 | 1 | 0.14 | N/A |
| medium containing sperm (cultured 4–8 h) | 100 | 1 | 0.22 | N/A |
| medium containing sperm (cultured 4–8 h) | 3 | 2 | 0.13 | N/A |
| medium containing sperm (cultured 4–8 h) | 10 | 2 | 0.362 | N/A |
| medium containing sperm (cultured 4–8 h) | 50 | 2 | 0.254 | N/A |
| medium containing sperm (cultured 4–8 h) | 100 | 2 | 0.154 | N/A |
| medium containing sperm (cultured 4–8 h) | 3 | 3 | 0.166 | N/A |
| medium containing sperm (cultured 4–8 h) | 10 | 3 | 0.228 | N/A |
| medium containing sperm (cultured 4–8 h) | 50 | 3 | 0.168 | N/A |
| medium containing sperm (cultured 4–8 h) | 100 | 3 | 0.176 | N/A |
| medium containing sperm (cultured 4–8 h) | 3 | 4 | 0.142 | N/A |
| medium containing sperm (cultured 4–8 h) | 10 | 4 | 0.29 | N/A |
| medium containing sperm (cultured 4–8 h) | 50 | 4 | 0.248 | N/A |
| medium containing sperm (cultured 4–8 h) | 100 | 4 | 0.134 | N/A |
| medium containing sperm (cultured 1 d) | 3 | 1 | 0.142 | N/A |
| medium containing sperm (cultured 1 d) | 10 | 1 | 0.242 | N/A |
| medium containing sperm (cultured 1 d) | 50 | 1 | 0.118 | N/A |
| medium containing sperm (cultured 1 d) | 100 | 1 | 0.24 | N/A |
| medium containing sperm (cultured 1 d) | 3 | 2 | 0.118 | N/A |
| medium containing sperm (cultured 1 d) | 10 | 2 | 0.272 | N/A |
| medium containing sperm (cultured 1 d) | 50 | 2 | 0.114 | N/A |
| medium containing sperm (cultured 1 d) | 100 | 2 | 0.114 | N/A |
| medium containing sperm (cultured 1 d) | 3 | 3 | 0.164 | N/A |
| medium containing sperm (cultured 1 d) | 10 | 3 | 0.114 | N/A |
| medium containing sperm (cultured 1 d) | 50 | 3 | 0.14 | N/A |
| medium containing sperm (cultured 1 d) | 100 | 3 | 0.116 | N/A |
| medium containing sperm (cultured 1 d) | 3 | 4 | 0.118 | N/A |
| medium containing sperm (cultured 1 d) | 10 | 4 | 0.16 | N/A |
| medium containing sperm (cultured 1 d) | 50 | 4 | 0.14 | N/A |
| medium containing sperm (cultured 1 d) | 100 | 4 | 0.14 | N/A |
| medium containing sperm (cultured 3 d) | 3 | 1 | 0.27 | N/A |
| medium containing sperm (cultured 3 d) | 10 | 1 | 0.252 | N/A |
| medium containing sperm (cultured 3 d) | 50 | 1 | 0.34 | N/A |
| medium containing sperm (cultured 3 d) | 100 | 1 | 0.11 | N/A |
| medium containing sperm (cultured 3 d) | 3 | 2 | 0.256 | N/A |
| medium containing sperm (cultured 3 d) | 10 | 2 | 0.174 | N/A |
| medium containing sperm (cultured 3 d) | 50 | 2 | 0.168 | N/A |
| medium containing sperm (cultured 3 d) | 100 | 2 | 0.182 | N/A |
| medium containing sperm (cultured 3 d) | 3 | 3 | 0.156 | N/A |
| medium containing sperm (cultured 3 d) | 10 | 3 | 0.142 | N/A |
| medium containing sperm (cultured 3 d) | 50 | 3 | 0.152 | N/A |
| medium containing sperm (cultured 3 d) | 100 | 3 | 0.146 | N/A |
| medium containing sperm (cultured 3 d) | 3 | 4 | 0.164 | N/A |
| medium containing sperm (cultured 3 d) | 10 | 4 | 0.17 | N/A |
| medium containing sperm (cultured 3 d) | 50 | 4 | 0.112 | N/A |
| medium containing sperm (cultured 3 d) | 100 | 4 | 0.204 | N/A |
| medium containing sperm (cultured 5 d) | 3 | 1 | 0.13 | N/A |
| medium containing sperm (cultured 5 d) | 10 | 1 | 0.11 | N/A |
| medium containing sperm (cultured 5 d) | 50 | 1 | 0.17 | N/A |
| medium containing sperm (cultured 5 d) | 100 | 1 | 0.166 | N/A |
| medium containing sperm (cultured 5d) | 3 | 2 | 0.168 | N/A |
| medium containing sperm (cultured 5d) | 10 | 2 | 0.182 | N/A |
| medium containing sperm (cultured 5d) | 50 | 2 | 0.216 | N/A |
| medium containing sperm (cultured 5 d) | 100 | 2 | 0.456 | N/A |
| medium containing sperm (cultured 5 d) | 3 | 3 | 0.252 | N/A |
| medium containing sperm (cultured 5 d) | 10 | 3 | 0.17 | N/A |
| medium containing sperm (cultured 5 d) | 50 | 3 | 0.286 | N/A |
| medium containing sperm (cultured 5 d) | 100 | 3 | 0.228 | N/A |
| medium containing sperm (cultured 5 d) | 3 | 4 | 0.18 | N/A |
| medium containing sperm (cultured 5 d) | 10 | 4 | 0.192 | N/A |
| medium containing sperm (cultured 5 d) | 50 | 4 | 0.178 | N/A |
| medium containing sperm (cultured 5 d) | 100 | 4 | 0.13 | N/A |
| sperm injection zona pellucida | / | 1 | 0.186 | N/A |
| sperm injection zona pellucida | / | 2 | 0.308 | N/A |
| sperm injection zona pellucida | / | 3 | 0.114 | N/A |
| sperm injection zona pellucida | / | 4 | 0.134 | N/A |
| sperm injection zona pellucida | / | 5 | 0.146 | N/A |
| In vitro fertilization zona pellucida | / | 1 | 0.166 | N/A |
| In vitro fertilization zona pellucida | / | 2 | 0.186 | N/A |
| In vitro fertilization zona pellucida | / | 3 | 0.144 | N/A |
| In vitro fertilization zona pellucida | / | 4 | 0.12 | N/A |
| In vitro fertilization zona pellucida | / | 5 | 0.15 | N/A |
| In vitro fertilization zona pellucida | / | 6 | 0.178 | N/A |
| In vitro fertilization zona pellucida | / | 7 | 0.386 | N/A |
| Blank drop | / | 1 | 0.216 | N/A |
| Blank drop | / | 2 | 0.168 | N/A |
| Blank drop | / | 3 | 0.142 | N/A |
| Blank drop | / | 4 | 0.154 | N/A |
| Positive control 10 pg GM12878 gDNA | / | 1 | 15.8 | 46,XN |
| Positive control 10 pg GM12878 gDNA | / | 2 | 11.7 | 46,XN |

**Supplementary Table 3.** CNV results of SCM and TE in 27 donated embryos

| Sample ID | SCM | TE | Ploidy Concordance |
| --- | --- | --- | --- |
| 1PN-1 | N/A | 46,XY | -- |
| 1PN-2 | 46,XX, -6(×1,mos,~50%),+10(×3,mos,~50%) | 47,XX,+6(×3) | TP |
| 1PN-3 | 46,XX | 46,XX | TN |
| 1PN-4 | 46,XX, -16(×1,mos,~50%) | 45,XX, -16(×1) | TP |
| 1PN-5 | 46,XX, -4(×1,mos,~60%) | 46,XX | FP |
| 1PN-6 | 46,XX,+3(×3,mos,~50%),+7(×3,mos,~50%) | 46,XX | FP |
| 1PN-7 | 47,XX,+18(×3) | 45,XX, -Xp(×1,mos,~50%), -Xq(×1,mos,~50%), -18(×1) | TP |
| 1PN-8 | 46,XY | 46,XY | TN |
| 1PN-9 | 46,XX | 46,XX | TN |
| 1PN-10 | 48,XYY,+Y(×2),+3(×3,mos,~50%),+4(×3),  +11(×3), -13(×1), -14(×1),+15(×3),+17(×3), -18(×1), 20(×1),+21(×3) | 46,XY | FP |
| 1PN-11 | 47,XX,+16(×3) | 45,XX, -16(×1) | TP |
| 1PN-12 | 46,XY, -4(×1,mos,~50%),+8(×3,mos,~50%) | 46,XY | FP |
| 1PN-13 | 46,XX | 46,XX | TN |
| 1PN-14 | 46,XX, -3p(×1,mos,~40%),+15(×3,mos,~40%) | 45,XX, -3(×1) | TP |
| 1PN-15 | 46,XX | 46,XX | TN |
| 1PN-16 | 47,XY,+2(×3),+4(×3,mos,~50%),+11(×3,mos,  ~60%) | 46,XY | FP |
| 1PN-17 | 46,XX | 46,XX | TN |
| 1PN-18 | 45,X, -X(×1), -3(×1,mos,~60%),+6(×3,mos,~50%), 10(×1),+12(×3,mos,~60%),+18(×3),+21(×3,mos,~70%),+22(×3,mos,~50%) | 46,XX | FP |
| 1PN-19 | 45,XX, -4(×1,mos,~60%), -9(×1,mos,~60%),+14  (×3,mos,~50%), -21(×1) | 45,XX, -21(×1) | TP |
| 1PN-20 | 46,XY, -8p(×1,mos,~60%),+8q(×3,mos,~50%) | 46,XY | FP |
| 1PN-21 | 46,XY | 46,XY | TN |
| 1PN-22 | 46,XY | 46,XY | TN |
| 1PN-23 | 46,XY, -Yp(×0,mos,~50%), -Yq(×0,mos,~40%) | 46,XY | FP |
| 1PN-24 | 52,XX,+7(×5),+18(×5) | 51,XX,+7(×4),+18(×5) | TP |
| 1PN-25 | 46,XY | 46,XY | TN |
| 1PN-26 | 46,XY | 46,XY | TN |
| 1PN-27 | 46,XY | 46,XY | TN |

**Supplementary Table 4.** The overview of NGS results from SCM and TE in 27 donated 1PN embryos

| **Samples** | **SCM** | **TE** |
| --- | --- | --- |
| WGA success rate | 96.3% (26/27) | 100% (27/27) |
| Sex | 26 | 27 |
| XX | 57.7% (15/26) | 55.6% (15/27) |
| XY | 42.3% (11/26) | 44.4% (12/27) |
| Sex concordance rate | 100% (26/26) | |
| Ploidy | 26 | 27 |
| Euploid | 42.3% (11/26) | 74.1% (20/27) |
| Aneuploid | 26.9% (7/26) | 25.9% (7/27) |
| Mosacism | 30.8% (8/26) | 0% (0/27) |
| Ploidy concordance rate | 69.2% (18/26) | |

**Supplementary Table 5.** The CNV results, parental contamination and other information forms of 161 clinical IVF samples

**Supplementary Table 6.** Comparison of maternal contamination between fresh embryos and frozen embryos

| Embryos (n) | Maternal contamination ratio<30%, % (95% CI) | Maternal contamination ratio ≥30%, % (95% CI) | All, % (95% CI) |
| --- | --- | --- | --- |
| Fresh embryos (106) | 19.8 (13.3–28.4) | 16.0 (10.3–24.2) | 35.8 (27.4–45.3) |
| Frozen embryos (52) | 9.6 (4.2–20.6) | 9.6 (4.2–20.6) | 19.2 (10.8–31.9) |
| p value | 0.104 | 0.273 | 0.033 |

**Supplementary Table 7.** Clinical baseline characteristics of patients

| Characteristics | IVF | ICSI |
| --- | --- | --- |
| No. of cycles (patients) | 25 | 37 |
| Mean female age (SD), y | 35.7 (4.1) | 35.4 (5.1) |
| Mean male age (SD), y | 35.4 (4.9) | 36.7 (5.4) |
| Mean female BM I(SD), kg/m^2^ | 22.0 (3.0) | 21.9 (2.6） |
| Primary PGT-A indication | | |
| AMA | 15 | 22 |
| RIF | 1 | 3 |
| RPL | 2 | 6 |
| Mixed caused | 2 | 1 |
| Male factor infertility | 2 | 2 |
| Previous aneuploid conception | 3 | 3 |

**Supplementary Table 8.** The CNV results and other information forms of 122 ICSI samples

**Supplementary Table 9.**  Comparison of the diagnostic parameters of SCM from different blastocyst quality and different sampling times in 161 clinical IVF samples

|  | NICS amplification success rate, % (95% CI) | TE Amplification success rate, % (95% CI) | analyzed samples(n) | Concordance,% (95% CI) | Sensitivity,% (95% CI) | Specificity,% (95% CI) | PPV,% (95% CI) | NPV,% (95% CI) |
| --- | --- | --- | --- | --- | --- | --- | --- | --- |
|  |  |  |  |  |  |  |  |  |
| **Quality grade (n)** | | | | | | | | |
| Good (51) | 82.4 (69.7–90.4) | 98.0 (89.7–99.9） | Good (41) | 70.7 (55.5–82.4) | 81.8 (52.3–96.8) | 66.7 (48.8–80.8) | 47.4 (27.3–68.3) | 90.9 (72.2–98.4) |
| Fair (62) | 95.2 (86.7–98.7) | 96.8 (89.0–99.4) | Fair (57) | 71.9 (59.2–81.9) | 94.7 (75.4–99.7) | 60.5 (44.7–74.4) | 54.6 (38.0–70.2) | 95.8 (79.8–99.8) |
| Poor (48) | 95.8 (86.0–99.3) | 100.0 (92.6–100） | Poor (46) | 82.6 (69.3–90.9) | 92.9 (77.4–98.7) | 66.7 (43.8–83.7) | 81.3 (64.7–91.1) | 85.7 (60.1–97.5) |
| p value | 0.037 | 0.778 |  | 0.38 | 0.695 | 0.84 | 0.021 | 0.621 |
| **Day of blastocyst biopsy（n）** | | | | | | | | |
| D5 (21) | 81.0 (60.0–92.3） | 100.0 (84.5–100) | D5 (17) | 76.5 (52.7–90.4) | 100.0% (67.6–100) | 55.6% (26.7–81.1) | 66.7% (39.1–86.2) | 100.0% (56.6–100) |
| D6 (125) | 92.0 (85.9–95.6) | 98.4 (94.4–99.7) | D6 (113) | 75.2 (66.5–82.3) | 90.5% (77.9–96.2) | 66.2% (54.6–76.1) | 61.3% (48.9–72.4) | 92.2% (81.5–96.9) |
| D7 (15) | 100.0 (79.6–100) | 93.3% (70.2–99.7) | D7 (14) | 71.4 (45.4–88.3) | 87.5% (52.9–99.4） | 50.0% (18.8–81.2) | 70.0% (39.7–89.2) | 75.0% (30.1–98.7) |
| p value | 0.120 | 0.296 |  | 0.999 | 0.84 | 0.683 | 0.814 | 0.341 |

**Supplementary Table 10.** The TE results of 9 ‘Good’ samples

| Sample ID | TE CNV | NICS CNV | Gardner score | Quality grade |
| --- | --- | --- | --- | --- |
| 1-4 | 46,XN | N/A | 6AA | Good |
| 5-1 | 46,XN | N/A | 6AB | Good |
| 5-2 | 46,XN | N/A | 6BA | Good |
| 13-4 | 47,XN,+15(×3) | N/A | 5AB | Good |
| 14-3 | 46,XX,-X(×1,mos,~70%) | N/A | 6BA | Good |
| 14-4 | 46,XN | N/A | 6BA | Good |
| 14-9 | 46,XN | N/A | 6AA | Good |
| 15-1 | 46,XN | N/A | 4BA | Good |
| 15-2 | 46,XN | N/A | 6AA | Good |

**Supplementary Table 11.** The proportion of euploid embryos between the IVF and ICSI groups

| Fertilization method (n) | Euploid embryos (adjusted TE results) | | |
| --- | --- | --- | --- |
|  | Number | % (95% CI) | p value |
| IVF (158) | 94 | 59.5 (51.7–66.8) | 0.553 |
| ICSI (118) | 66 | 55.9 (46.9–64.6) |  |
